# The Transmission of Multidrug-Resistant Bacteria and Antibiotic Resistance Genes from the One Health Perspective

**DOI:** 10.3390/antibiotics15100980

**Published:** 2026-10-04

**Authors:** Keke Chai, Chenhui Xu, Gaojie Cao, Linxi Wang, Yuying Chen, Beibei Wu

**Affiliations:** 1 School of Public Health, Wenzhou Medical University, Wenzhou 325035, China; chaikeke0119@163.com; 2 School of Laboratory Medicine and Life Science, Wenzhou Medical University, Wenzhou 325035, China; xuchenhui0521@163.com (C.X.); caogaojie0111@163.com (G.C.); 3 School of Public Health, Hangzhou Medical College, Hangzhou 310051, China; wanglx9923sjk@163.com (L.W.); chenyuying0731@163.com (Y.C.); 4 Zhejiang Key Lab of Vaccine, Infectious Disease Prevention and Control, Zhejiang Provincial Center for Disease Control and Prevention, Hangzhou 310051, China

**Keywords:** antimicrobial resistance, multidrug resistance, antibiotic resistance genes, One health

## Abstract

Antimicrobial resistance (AMR) is a complex One Health problem involving people, non-human species, and the environment. This review examines the distribution and transmission of multidrug-resistant (MDR) bacteria and antibiotic resistance genes (ARGs) across these domains. One Health has broken the long-standing isolation of MDR research by establishing a unified cross-disciplinary analytical framework, supporting interdisciplinary MDR studies, and systematically mapping the complete transmission chains and pathways of MDR determinants (including drug-resistant pathogens and resistance genes) at the human–animal–environment interface. This holistic perspective has become an indispensable guiding principle for addressing complex global health challenges.

## 1. Introduction

AMR now poses a grave threat to global public health. According to global burden estimates, in 2019, approximately 1.27 million deaths were directly attributable to bacterial AMR, representing about 2% of global all-cause mortality, while 4.95 million deaths were associated with AMR, including the attributable deaths [1]. If effective interventions are not implemented, by 2050, AMR is projected to be associated with 8.22 million deaths annually, including 1.91 million deaths directly attributable to resistance [2]. However, sustained antibiotic selective pressure and enhanced gene exchange, including horizontal gene transfer (HGT) mediated by plasmids, transposons, or integrons, have driven adaptive evolution and the accumulation of multiple resistance determinants. This trajectory has given rise to MDR. MDR significantly narrows the therapeutic options available for clinical treatment and complicates infection control. In addition, multidrug-resistant microbes and resistance genes can be widely spread at the human–animal–environment interface through food chains and environmental components (e.g., wastewater, soil). This transboundary transmission pattern transcends traditional domain boundaries, exacerbating the complexity of public health risks posed by MDR and highlights the interdependence of health across human, non-human species, and environmental domains. In light of this dynamic transboundary transmission and the interconnected nature of MDR risks, isolated interventions confined to a single domain are inherently inadequate to curb the global spread of MDR bacteria and ARGs. This highlights the necessity of adopting the One Health approach, an integrated, holistic framework that recognizes the intrinsic interdependence between human health, non-human species health, and the integrity of ecological systems. However, current research and interventions in these domains remain fragmented, which limits comparable surveillance, source attribution, and coordinated action. To address this fragmentation, this review focuses on the transmission dynamics of MDR bacteria and ARGs from a One Health perspective, aiming to synthesize current research progress and provide insights for future interdisciplinary governance.

## 2. Review Methodology

This narrative review was based on the peer-reviewed literature available through 2026 identified in PubMed, Web of Science, and Scopus. The core search combined terms for AMR, MDR, MDR organism, or ARG with One Health, human, non-human species, livestock, companion animal, soil, river, wastewater, and transmission. Titles and abstracts were screened for relevance to human, non-human species, or environmental reservoirs and to evidence on occurrence, selection, or transmission. Full texts were then assessed for study design, setting, genomic or epidemiological evidence, and limitations. Peer-reviewed studies were prioritised, with the recent literature supplemented by foundational older studies.

## 3. Terminology and Scope

In this review, AMR is used as the overarching term for reduced susceptibility to antimicrobial agents, irrespective of whether resistance is intrinsic or acquired. MDR is a phenotypic category and is defined as resistance to at least one agent in three or more antimicrobial categories; intrinsic resistance and genes that are present but not expressed should not be equated with acquired MDR [3]. An MDR organism refers to an organism with an MDR phenotype, whereas ARG refers to a genetic determinant. The presence of an ARG does not necessarily indicate phenotypic resistance, and only ARGs associated with mobile genetic elements have the potential for horizontal transfer. This distinction is maintained throughout the review. MDR emerges from interactions among microbial biology, antimicrobial exposure, ecological connectivity, and human activities [3,4].

Gram-negative bacteria inherently possess a prominent natural defense barrier, primarily attributed to the low permeability of their outer membrane and the constitutive expression of efflux pumps, which collectively confer intrinsic resistance to numerous antimicrobial agents [5]. Studies of bacteria in the family Hafniaceae found intrinsic resistance to colistin and high genetic diversity, indicating that resistance is not restricted to a small number of clones [6]. Cellular structure and genetic composition jointly determine intrinsic resistance: lipopolysaccharides block hydrophobic molecules, porins restrict the entry of hydrophilic molecules above approximately 700 Da, and efflux systems remove some agents that enter the cell [5].

Beyond such intrinsic mechanisms, the development of MDR in bacteria is predominantly driven by two core genetic pathways: *de novo* mutations and HGT (formerly referred to as bacterial recombination). Specifically, *de novo* mutations encompass genetic alterations such as point mutations, insertions, deletions, or gene duplications that modify the function of drug targets or related regulatory elements; in contrast, HGT denotes the interbacterial transfer of resistance-related genetic material (e.g., plasmids, transposons, integrons) via non-vertical transmission mechanisms (e.g., conjugation, transformation, transduction) [4]. Accumulating genomic sequencing studies have further confirmed that bacteria harbor multiple efflux pump systems, and the synergistic activation or overexpression of distinct efflux pumps is a key driver of MDR, as this collaborative effect significantly augments bacterial tolerance to a broad spectrum of structurally and functionally divergent antibiotics. Furthermore, while the formation of biofilms is not a direct genetic mechanism underlying MDR, it can nonetheless influence drug resistance. Studies have shown that in clinically isolated *Staphylococcus epidermidis* strains, a strong biofilm-forming ability is associated with β-lactam antibiotic resistance and MDR; strains capable of forming robust biofilms exhibit higher levels of drug resistance [7].

## 4. The One Health Concept

The concept of One Health originated in 1964, when Calvin Schwabe proposed “One Medicine”, advocating for integrated practices and research across human and veterinary medicine to address zoonotic diseases [8]. By breaking the disciplinary barriers that have long separated human and non-human species health domains, Schwabe’s “One Medicine” laid a critical theoretical and practical foundation for recognizing the interdependence of health outcomes across species boundaries, and provided an initial analytical paradigm for subsequent cross-disciplinary health research. With the emergence of 21st-century global challenges—such as AMR and climate change—the One Health framework was expanded to incorporate the health of plants, soils, and entire ecosystems. It highlights the interconnected health of all organisms via the cycling of microbial communities in ecological systems [9]. This holistic perspective has since become an indispensable guiding principle for addressing complex global health issues, particularly in the context of studying the distribution and transmission of MDR organisms across the human–animal–environment interface.

Now, the One Health concept has evolved from an interdisciplinary perspective into a globally recognized framework for addressing complex health challenges. In a joint definition released in 2021 by the World Health Organization (WHO), the Food and Agriculture Organization (FAO), the World Organization for Animal Health (WOAH, formerly OIE), and the United Nations Environment Programme (UNEP), One Health is characterized as an integrated and unifying approach. It seeks to sustainably balance and optimize the health of humans, non-human species, and ecosystems, grounded in the recognition of their fundamental interconnections [10]. The One-Health approach emphasizes interdisciplinary collaboration across academic disciplines and transdisciplinary engagement between academia and society. It focuses on addressing health issues at the human–animal–environment interface, with the goal of delivering benefits that are unattainable when these distinct sectors operate in isolation [11].

## 5. Distribution of MDR in Three Domains

### 5.1. Human

In the human domain, MDR organisms are concentrated in acute-care hospitals, long-term care facilities (LTCFs), and community settings. Their prevalence and strain composition are shaped by antimicrobial prescribing, healthcare access, infection-control capacity, and socioeconomic conditions. For instance, a nationwide investigation conducted in the United States in 2022 estimated that approximately 570,000 cases of drug-resistant infections occurred across the country, among which 77% were community-onset infections and the remaining 23% were from hospital-associated environments [12]. Notably, this surveillance also identified multidrug-resistant *Staphylococcus aureus* (MRSA) and extended-spectrum β-lactamase-producing *Klebsiella pneumoniae* as the most predominant MDR pathogens driving these infection events, highlighting their critical role in both healthcare-associated and community-onset AMR burdens.

In the human health domain, clinical settings, especially acute-care hospitals and LTCFs, are the most concentrated and high-risk reservoirs of multidrug-resistant pathogens, owing to frequent antimicrobial exposure, close contact among vulnerable populations, and repeated transfer of patients between healthcare units. Epidemiological surveillance targeting adult healthcare-associated infections has further characterized the pathogen profile of these settings: *Escherichia coli* (18%), *Staphylococcus aureus* (12%), and *Klebsiella* spp. (9%) have been identified as the three most commonly reported etiological agents [13]. A noteworthy observation from this surveillance is that the overall burden of MDR pathogens in long-term acute care hospitals is substantially higher than that in general hospital wards, a disparity likely driven by prolonged hospitalization, frequent use of broad-spectrum antimicrobials, and a higher proportion of immunocompromised patients in specialized long-term care settings. LTCFs, in particular, function as critical hotspots for the silent transmission of MDR pathogens, given their high-density living environments and the aggregation of residents with weakened immune function, chronic comorbidities, and a history of repeated antimicrobial treatment. Empirical evidence for this transmission risk comes from a cross-sectional study conducted in Amsterdam, the Netherlands, which enrolled 385 residents from 12 LTCFs and detected an overall MDR Gram-negative bacilli carriage rate of 18.2% [14]. Among these carriers, extended-spectrum β-lactamase-producing Enterobacteriaceae accounted for 14.5% of cases—a prevalence significantly exceeding that reported in the general population. The Amsterdam study also highlighted two key concerns: first, there were substantial variations in MDR carriage rates among different LTCFs, suggesting that facility-specific infection control measures and antimicrobial stewardship policies may play a decisive role in shaping local transmission dynamics; second, routine clinical culture methods failed to identify 80% of these carriers, indicating that a large number of asymptomatic carriers remain undiagnosed in such settings and may serve as unrecognized reservoirs for cross-transmission to other residents and healthcare workers.

The community MDR pathogens are mainly Gram-negative bacteria. The drug resistance mechanisms of *Pseudomonas aeruginosa*, *Acinetobacter baumannii*, *Klebsiella pneumoniae* and *Stenotrophomonas maltophilia* include β-lactamase production, efflux pump activation and membrane permeability alteration [15]. A 10-year (2012–2022) genomic epidemiological study in Kenya, Uganda, and Jordan revealed that multidrug-resistant *Escherichia coli* in community populations belonged to the ST131 sequence type clone, while *Klebsiella pneumoniae* was mainly composed of clones such as ST14 and ST147 [16]. Furthermore, community-associated methicillin-resistant Staphylococcus aureus (CA-MRSA) has become a major pathogen causing severe invasive infections in the community population. For example, studies indicate that in the United States, the 18-IV sequence clone (ST8-IV), known as USA300, has become highly prevalent, outpacing methicillin-sensitive Staphylococcus aureus (MSSA) and other MRSA strains in both community and hospital settings [17].

### 5.2. Environment

AMR phenotypes and their corresponding ARGs are recognized as ubiquitous environmental elements, with mounting evidence indicating that the majority of resistance determinants of human pathogenic bacteria have evolutionary origins in the intrinsic microbial communities of natural ecosystems [18]. From a microbial ecological perspective, the environmental domain, an indispensable component of the human–animal–environment interface, can be categorized into two primary subtypes: the natural environment, including aquatic ecosystems (e.g., rivers, lakes, and groundwater) and terrestrial ecosystems (e.g., soil and sediment), and the built environment, a human-modified matrix that includes wastewater treatment plants (WWTPs), agricultural production sites, aquaculture facilities, and clinical settings [19]. Considering the critical role of environmental reservoirs in mediating the dissemination of MDR organisms and ARGs across ecological boundaries, this review focuses on four representative environmental matrices: soil, riverine systems, raw sewage, and wastewater treatment plants, as these habitats are key hotspots for MDR proliferation, gene exchange, and cross-domain transmission.

Armed conflicts and weapon contamination may also add environmental selection pressures. Conflicts damage environmental sanitation, wastewater treatment systems, and medical infrastructure. Furthermore, weapons and explosives may release metals, explosive residues, and other pollutants into soil and water. These pollutants may, through the mechanisms of co-resistance, cross-resistance, and the action of mobile genetic elements, collectively promote the development of antimicrobial drug resistance [20,21].

#### 5.2.1. Soil

A growing number of metagenomic and culture-based studies have unequivocally identified the soil environment as a primary natural reservoir and evolutionary cradle of ARGs, with soil metagenomes harboring the broadest diversity of transferable antibiotic resistance gene determinants reported to date [22,23]. Empirical evidence for the ancient and intrinsic nature of soil-borne resistance comes from a landmark investigation of microbial communities in Lichugila Cave, New Mexico, USA, an oligotrophic and isolated ecosystem where microorganisms have evolved in the absence of anthropogenic antibiotic exposure for over 4 million years. Notably, the cave-dwelling microbial strains exhibited robust resistance to multiple clinically relevant antibiotics, with certain isolates demonstrating cross-resistance to up to 14 commercial antimicrobial agents [24]. This finding provides compelling proof that ARGs are not merely anthropogenically driven contaminants but instead represent ancient, evolutionarily conserved elements of the environmental resistome. Further reinforcing the link between soil resistomes and clinical MDR organisms, molecular characterization studies have revealed that the nucleotide sequences of ARGs from soil bacteria—conferring resistance to five major antibiotic classes including β-lactams, aminoglycosides, sulfonamides, amphenicols, and tetracyclines—can share high sequence identity with determinants in a wide range of clinically relevant human pathogens [25]. This sequence homology is consistent with the phenomenon of historical or current HGT across the environmental–clinical interface, supporting the hypothesis that soil can serve as a source of determinants of bacterial drug resistance.

#### 5.2.2. River

As critical hubs connecting terrestrial, agricultural, and urban ecosystems, rivers are primary sources of water for agricultural irrigation and domestic consumption, while simultaneously functioning as pivotal conduits for the dissemination of MDR organisms and their ARGs across the human–animal–environment interface. Jiang et al. [26] reported that the abundance and diversity of ARGs in waterborne bacteria were significantly higher than those in other habitats. Metagenomic studies of the Beijing Xinfeng River revealed the highest abundance of quinolone resistance genes in both the water and sediment, along with the detection of 59 human pathogenic bacteria, 69 mobile genetic elements, and 19 virulence factors [27]. River ecosystem resilience can be affected by sewage inflow and human activities [28]. Studies have confirmed that unregulated wastewater discharge significantly alters the bacterial colony structure and resistance profile of specific rivers [29]. Human activities can promote the spread of resistance genes in rivers [30,31]. Furthermore, studies have confirmed that a significant proportion of ARGs shared between human intestines and pathogenic bacteria in rivers are associated with mobile genetic elements. Although fecal contamination from human activities can facilitate the spread of ARGs to environmental resistance populations, it is the mobile resistance genes, rather than the drug-resistant fecal bacteria themselves, that actually proliferate in the environment [31].

#### 5.2.3. Wastewater and WWTP

WWTPs are reservoirs of antibiotic-resistant bacteria and ARGs, and their environmental characteristics provide favorable conditions for the spread of antimicrobial resistance. WWTPs receive large volumes of sewage from urban, industrial, and hospital sources. Multiple ARGs can be detected in both the influent and effluent of these plants. During the treatment process, these ARGs may undergo further enrichment and dissemination, and may even be released into the natural environment through sludge [32]. In wastewater treatment plants, the primary drug resistance genes are those conferring resistance to macrolides (*ermB*, *ermF*), tetracyclines (*tetW*, *tetA*, *tetC*), sulfonamides (*sul1*, *sul2*), and β-lactams (*bla*OXA, *bla*TEM) [33]. Metagenomic sequencing of the influent and effluent from 12 international wastewater treatment plants found that the most abundant ARG classes conferred multidrug, macrolide-lincosamide-streptogramin (MLS), β-lactam, tetracycline, and aminoglycoside resistance [34]. Hospital wastewater is of particular concern because it combines concentrated antimicrobial use with a high load of resistant bacteria and mobile genetic elements. Conventional collection and pretreatment do not reliably eliminate these determinants. Studies have indicated that the predominant bacterial strains in hospital wastewater include Enterobacteriaceae, such as *Escherichia coli* and *Klebsiella pneumoniae*; *Acinetobacter* spp., such as *Acinetobacter baumannii*; *Pseudomonas* spp., such as *Pseudomonas aeruginosa*; and *Staphylococcus* spp., such as *Staphylococcus aureus* [35,36,37].

### 5.3. Non-Human Species

Antibiotics are used in non-human species for three main purposes: growth promotion, infection prevention, and clinical treatment. Regulatory policies vary significantly across different regions: in the European Union and several other jurisdictions, the use of antimicrobial agents to promote growth has been explicitly prohibited; in contrast, in other regions, this practice is still permitted or is subject to less stringent regulation, thereby creating distinct selective pressures [38,39,40]. Studies have demonstrated that MDR in Salmonella non-typhi in developed countries primarily stems from the inappropriate use of antibiotics in food-producing non-human species (e.g., cattle, poultry, and pigs). Drug-resistant strains are transmitted to humans through the food chain, with S*almonella Typhimurium* DT104 being a typical representative strain that has become prevalent in food-producing animals and humans in multiple countries worldwide [41]. Livestock farming is the main factor contributing to MDR in non-human species populations. A controlled trial demonstrated that after feeding pigs with growth-promoting antibiotics containing chlortetracycline, sulfadiazine, and penicillin, their gut microbiota underwent significant changes within 14 days. The proportion of Proteobacteria (predominantly *Escherichia coli*) increased from 1% to 11%, which was significantly higher than that in pigs not fed with antibiotics [42]. This study indicates that subtherapeutic antibiotic exposure in livestock was associated with enrichment of resistance determinants in the gut microbiota and may create selective conditions favouring resistance. Subsequent studies investigating antibiotic resistance in farm and slaughterhouse workers exposed to non-human species revealed that practitioners in the pork production chain (pig farmers and slaughterhouse workers) exhibited a higher total abundance of ARGs in feces compared to poultry farmers and the general population. Tetracycline, β-lactam, and macrolide resistance gene clusters were the predominant dominant resistance genes [43]. These findings suggest that occupational exposure to livestock could serve as a route for the transmission of resistance genes; however, it remains unclear whether the transmission of resistance determinants from non-human species to humans is directional or how widespread such transmission actually is.

In addition, companion animals and humans share the same living environment, and the close relationship and interaction between them enable the transmission of zoonotic diseases and antimicrobial-resistant strains between the two host groups [44]. A case–control study carried out in Germany from 2019 to 2022 indicated the potential for MDR organism transmission between companion animals (dogs, cats) and humans, although the actual incidence was extremely low (only 0.3% of human–animal pairs tested positive for pathogenic bacteria with identical genotypes) [45]. Another study indicates that most samples from pets and humans exhibit genetic differences; for a limited number of isolate pairs, cgMLST identified fewer allele differences, indicating greater genomic similarity and compatibility with recent transmission or a common source [46].

### 5.4. Interpreting Evidence for Transmission

**Figure 1:**
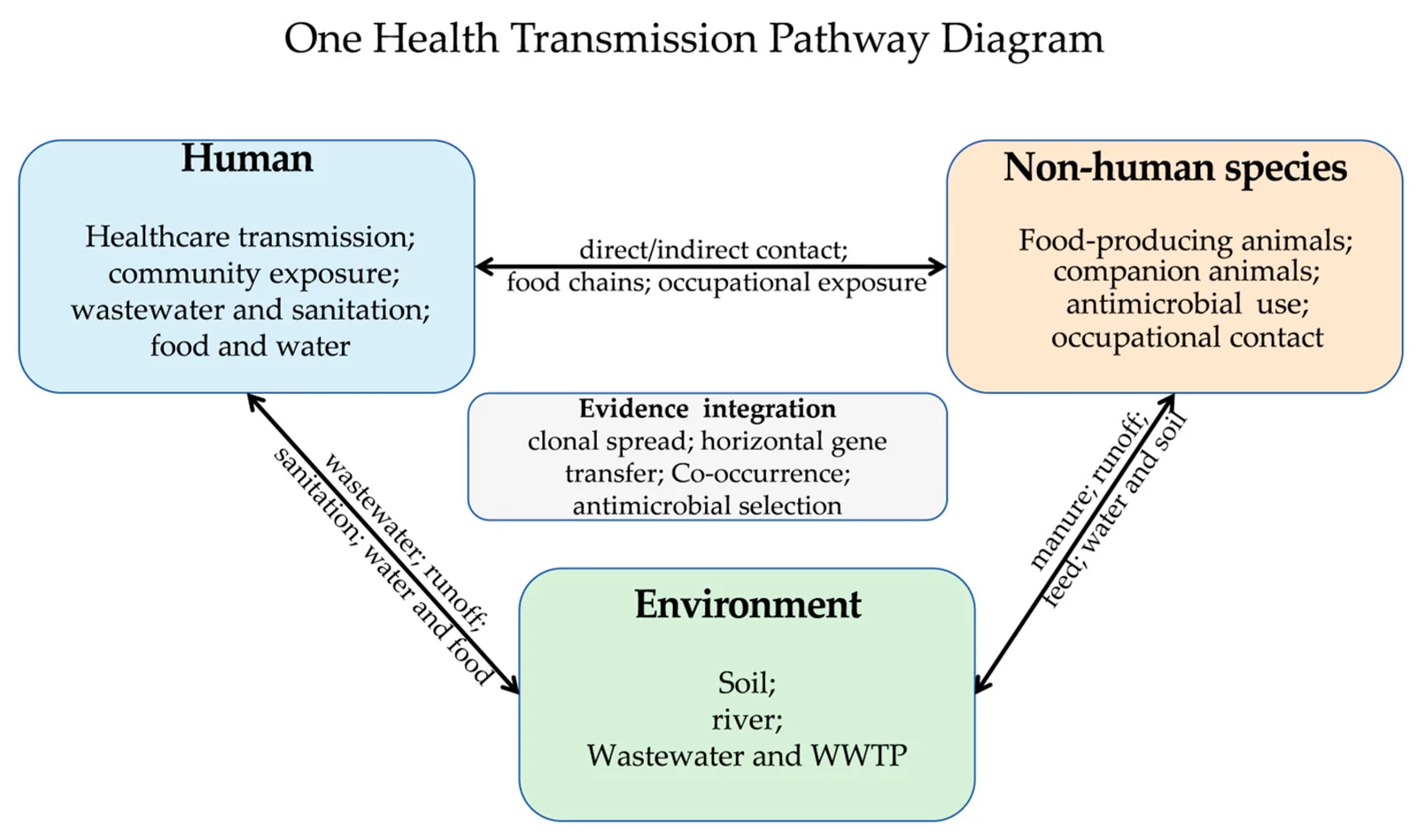
Conceptual framework for interpreting One Health transmission evidence.

The repeated detection of a resistance gene in human, non-human species, and environmental samples indicates co-occurrence or connectivity, but it is not by itself proof of direct transmission [47,48]. These processes are not mutually exclusive and may operate at different spatial and temporal scales (Figure 1).

Clonal dissemination refers to the transmission of the same drug-resistant bacterial strain or highly similar strains from one host to another host, or from one epidemiological domain to another. The strongest evidence for clonal dissemination is obtained when isolates from different sources demonstrate high genetic relatedness upon analysis using WGS or cgMLST methods, and these isolates are also associated in time-series and epidemiological data. For cgMLST, fewer allelic differences indicate greater genomic similarity and are compatible with recent transmission or a common source; a larger number of differences argues against recent clonal transmission [46,49,50]. Evidence for horizontal gene transfer requires characterisation of the genetic context, including flanking sequences, plasmids, transposons, integrons, or phages, together with evidence of mobility [51,52]. Environmental science and metagenomic studies are particularly effective for detecting mobile genetic elements and shared antibiotic resistance genes; however, such studies typically only indicate the existence of gene flow without being able to determine its direction [31,53,54]. Therefore, validation should be conducted by comprehensively integrating multi-dimensional information—including genomic similarity, the genetic context of mobile genetic elements, sampling time, exposure assessment, and epidemiological associations [49,50,53]. It is particularly important to note that an increase in the abundance of ARGs following exposure to antimicrobial agents indicates the presence of natural selection within the population [42,43]; however, whether such genes can be transferred to other hosts or tissues requires further verification.

Table 1 summarises representative studies and the strength of evidence for transmission across human, non-human animal, and environmental domains. Healthcare and long-term care data provide strong evidence for person-to-person and facility-associated spread of MDR bacteria [14]. Food-chain and occupational studies support plausible exposure routes but often cannot exclude reverse or common-source exposure [41,43]. Wastewater, river, soil, and companion animal studies show shared determinants and varying degrees of genomic similarity, yet many remain consistent with co-occurrence, historical gene exchange, or context-dependent transfer rather than contemporaneous cross-domain transmission [26,31,45,46,54].

**Table 1:** Representative Research Summary.

Study	Key Methods or Setting	Main Finding	Source DOI
Wolford et al., 2025 [12]	United States hospitalized AMR surveillance	574,000 hospitalizations; 77% community-onset	https://doi.org/10.1001/jamanetworkopen.2024.62059
van Dulm et al., 2019 [14]	Long-term care facilities; cross-sectional	18.2% multidrug-resistant Gram-negative carriage; many carriers undetected	https://doi.org/10.1371/journal.pone.0222200
Forsberg et al., 2012 [25]	Soil resistome compared with clinical pathogens	Shared resistance determinants and sequence homology	https://doi.org/10.1126/science.1220761
Lee et al., 2020 [31]	Human gut and river mobile resistome	Mobile ARGs shared across domains	https://doi.org/10.1186/s40168-019-0774-7
Looft et al., 2012 [42]	Controlled in-feed antibiotic trial in pigs	Gut microbiome and resistance-gene shifts after exposure	https://doi.org/10.1073/pnas.1120238109
Genath et al., 2024 [46]	Human-pet isolates; cgMLST	Most isolates distinct; few closely related pairs	https://doi.org/10.1186/s13756-024-01457-7
Wang et al., 2024 [51]	Inter-plasmid transfer experiments	Plasmid-mediated ARG transfer among pathogens	https://doi.org/10.1093/ismejo/wrad032
Feng et al., 2025 [54]	Regional multi-source metagenomics and culture	Plasmid/phage-mediated HGT and clonal expansion suggested	https://doi.org/10.1186/s40168-024-01983-x
de Koster et al., 2024 [55]	Human, pig and broiler One Health surveillance	Local and cross-regional circulation; no human-to-pig evidence	https://doi.org/10.1371/journal.pone.0298096

## 6. The Value of One Health in MDR Research

### 6.1. Breaking the Barriers of Traditional Single-Field Research in MDR

MDR poses a cross-disciplinary public health challenge that transcends the boundaries of individual research and application fields. Controlling MDR cannot be achieved through fragmented, domain-specific investigation paradigms or intervention strategies. Historically, MDR research has been confined to isolated domains: clinical microbiology and medical studies primarily focus on detecting, characterizing, and managing MDR pathogens isolated from human patients; veterinary research prioritizes monitoring and controlling AMR in livestock and companion animals, particularly in the context of agricultural antibiotic use; environmental science concentrates on the occurrence, distribution, and transmission of antibiotic residues and ARGs in soil, water bodies, and other ecosystems. A systems-based reasoning framework is therefore required, explicitly acknowledging the ecological and epidemiological interdependencies among human health, non-human species health, and environmental integrity. For instance, a One Health study that examined MRSA isolates for multidrug efflux systems revealed that MDR Staphylococcus aureus with active efflux systems is circulating across different One Health domains [56].

### 6.2. Expanding Research on MDR Transmission and Evolution

Traditional MDR research has been confined to single-environment resistance mechanisms, overlooking the interactions and connections between humans, non-human species, and the environment. One Health expands mechanistic studies by exploring cross-domain evolutionary dynamics, revealing how MDR spreads and evolves through interactions in different ecological niches. For instance, a One Health study conducted in Belgium and the Netherlands focused on colistin-resistant *Enterobacteriaceae* (ColR-E), covering multiple host groups including humans, pigs, and broilers. The research revealed that ColR-E is widely distributed across different domains, with antibiotic resistance rates showing cross-border and cross-host variations. Notably, the use of colistin in pig farms was associated with the development of colistin resistance. Genetic analysis confirmed local transmission, cross-regional spread and sustained internal circulation within farms, but no evidence of human-to-pig cross-host transmission was observed [55]. Other studies have shown that plasmids carrying multidrug-resistant genes (such as β-lactam resistance genes like *bla*_CTX-Ms_ and quinolone resistance genes like *qnr*) may move between soil bacteria, livestock gut microbiota, and human pathogens, potentially broadening resistance phenotypes [51,52].

### 6.3. Enhancing MDR Monitoring and Risk Early Warning

The escalating global threat posed by AMR has made it imperative to develop comprehensive monitoring strategies that transcend the boundaries of traditional clinical surveillance paradigms. Although next-generation sequencing technologies, especially whole-genome sequencing (WGS), have revolutionized AMR surveillance, their current applications remain disproportionately concentrated on clinical isolates. This narrow focus has led to the neglect of two critical reservoirs of antimicrobial resistance, the environment and non-human species populations, resulting in substantial gaps in the understanding of AMR evolutionary and transmission dynamics across interconnected ecosystems.

Against this background, a relevant study [53] has underscored the urgent necessity of integrating WGS and shotgun metagenomic sequencing technologies into the One Health framework to establish a unified genomic surveillance system for AMR. This integrated genomic approach is poised to significantly enhance the precision of detection and real-time tracking of resistance determinants, thereby shifting AMR management from a passive, reactive response model to a proactive, preventive control paradigm. However, the realization of this surveillance vision hinges on three core prerequisites: global standardization of sequencing methodologies to ensure cross-regional data comparability, unification of bioinformatics analysis workflows to eliminate interpretive discrepancies, and strengthening of cross-agency and cross-domain collaboration to translate surveillance data into timely and targeted interventions against emerging AMR threats.

Together, these genomic and data-platform developments provide the infrastructure needed to compare MDR determinants and transmission signals across human, non-human animal, and environmental domains. They also support integration of clinical, environmental, and non-human species-derived AMR surveillance data.

A One Health study in Dengfeng, Henan Province, China, combined metagenomic sequencing and resistant strain culture to conduct an in-depth analysis of 592 multi-source samples from human populations, food, and the environment. A total of 743 ARGs belonging to 40 categories were identified, with multidrug resistance genes dominating among all ARG types. This study reported that ARG loads in food and environmental samples were significantly higher than those in human samples and found associations between ARG abundance and dietary patterns or occupational exposure. Meanwhile, *Enterobacteriaceae* strains were identified as the core reservoirs and transmission vectors of regional ARGs. Further analyses suggested that plasmid- and phage-mediated horizontal gene transfer, together with clonal expansion of strains, may contribute to cross-interface ARG spread in the region. Flies and food were identified as important potential sources of ARGs input for human populations. Additionally, the OXA-347 carbapenemase gene carried by non-*Enterobacteriaceae* strains in the human gut, due to its high mobility, needs to be incorporated into key monitoring categories. Notably, machine learning models constructed based on microbiome profiles can achieve accurate prediction of carbapenem-resistant strains, which provides a novel technical solution for the technological upgrading of MDR surveillance and early warning systems [54]. A regional case study illustrates how integrated sampling can reveal cross-domain ARG flow rather than merely co-occurrence.

### 6.4. An Integrated One Health Evidence Framework

One Health does not simply combine three separate domains; rather, it enables the integration of research questions across host-, strain-, genetic-, and exposure-level dimensions [53,54,57]. Through such integrative research, the source, transmission pathways, and transmission mechanisms can be identified, thus facilitating the effective implementation of targeted interventions. However, integrating the three domains requires the fulfillment of the following conditions.

First and foremost, standardized sampling methods and metadata are required, as variations in sample type, sampling time, storage conditions, and metadata integrity can give rise to apparent discrepancies across different research fields [53,58]. Second, when using comparable antimicrobial susceptibility testing (AST) methods, any changes to the antimicrobial testing panel, testing methods, or breakpoint versions may lead to the reclassification of isolates, thereby rendering comparisons impossible [3,59]. Furthermore, whole-genome sequencing with unified quality control standards, as well as cgMLST or SNP analysis, is also crucial. These methods enable the effective differentiation between recent clonal dissemination and historical common ancestry relationships, and support meaningful comparisons across different studies [50]. Metagenomics, however, enables the capture of drug resistance determinants and facilitates comparative analysis of drug resistance genomes across human, non-human species, and environmental contexts [60,61]. Epidemiological metadata converts genomic and drug resistance phenotype data into testable transmission evidence by providing sub-attributes such as host, time, location, contact, and exposure [62]. Furthermore, adopting the FAIR+E metadata standard ensures that genomics and epidemiology data possess discoverability, interoperability, reusability, and comparability across different institutions and disciplines [63].

## 7. Conclusions

The currently most supported transmission routes for MDR bacteria and ARGs include: transmission within human healthcare settings, exposure through the food chain or occupational exposure, direct contact with companion animals, and exposure to wastewater or contaminated environmental media. However, current research still has gaps, including: lack of standardization in sampling across three domains, AST and genomic methodologies; insufficient direct evidence for cross-domain transmission; incomplete epidemiological metadata; and inadequate studies on the effectiveness of interventions, among others.

Addressing these knowledge gaps requires conducting One Health research that seamlessly integrates genomic, phenotypic, exposure-related, and epidemiological evidence. Furthermore, One Health surveillance enables the transformation of isolated, domain-specific observational data into comparable, practically applicable evidence for pathogen source tracking, transmission pathway inference, and risk assessment. In addition, it provides guidance for developing targeted intervention measures.

## Data Availability

No data was used for the research described in the article.
